# Lateral malleolar fractures Weber Type A and B: does percutaneous intramedullary screw confer a solid alternative to the traditional neutralization plate?

**DOI:** 10.1007/s00264-022-05425-x

**Published:** 2022-05-16

**Authors:** Sherif Hamdy Zawam, Mohamed Goda Mabrouk, Mahmoud Ahmed El-Desouky

**Affiliations:** Department of Trauma and Orthopedics, Faculty of Medicine, Cairo University, Giza, Egypt

**Keywords:** Ankle fractures, Lateral malleolar fractures, ORIF, Intramedullary screw fixation

## Abstract

**Purpose:**

To compare the clinical results, complication rates, and radiographic outcome between both methods of fixation of lateral malleolar fractures: lateral neutralization plates and intramedullary fully threaded screws.

**Patients and methods:**

This prospective case series study involved 73 patients with fractured lateral malleolus of type A, B according to Weber classification, to whom internal fixation was performed by either lateral plate and screws construct (Group A) or intramedullary screw (Group B). All patients were followed up for 12 months at least, with an average follow-up time of 12.7 months.

**Results:**

There was no significant difference in the functional outcome score between both groups. The intramedullary screw group had a significantly shorter operative time and time to full union (*P*<0.001 and =0.006 respectively). There was a relatively higher accuracy of reduction with the plate fixation group, but it was statistically insignificant. There was a relatively fewer complication rate with the use of intramedullary screw fixation compared to plate fixation.

**Conclusion:**

The use of intramedullary fixation is a good alternative for plate fixation in low fibular fractures (Weber A and B). Although plate fixation provides an optimal anatomic reconstruction of the fractures, intramedullary fixation may have a lower risk of complications.

## Introduction

Ankle malleolar fractures (Pott’s fractures) are among the commonest fractures in orthopedics, especially in old ages. They represent about 1/10 of all fractures [1, 2]. Uni-malleolar fractures represent about 68%, bi-malleolar fractures 25%, and 7% are tri-malleolar fractures [2].

Open reduction and internal fixation (ORIF) with plate and screws remains the gold standard for surgical management of lateral malleolar fractures [3].

However, ORIF with plate fixation may lead to several complications especially in old ages, patients with diabetic neuropathy, or patients with poor skin conditions [4, 5]. The incidence of complications may reach up to 30% as documented in certain studies [6]. Complaints regarding prominent hardware may reach up to 50% of the patients, wound problems are present in up to 26%, and implant failure may occur in 14% [7–9].

Therefore, intramedullary fixation of the fibula appeared as an alternative method. It was first done using Inyo nails and then modified using more advanced nails and screws technologies [10, 11]. The main advantage is that fixation is done through small incisions with little soft-tissue dissection [12, 13].

Therefore, the choice of the proper method of fixation was the main concern in several studies on malleolar ankle fractures. The aim of our study was to evaluate whether intramedullary fixation of lateral malleolar fractures using a 3.5-mm screw is comparable to the traditional lateral plate fixation method.

## Materials and methods

This study was conducted on 80 patients who presented to the emergency department of a tertiary trauma centre with lateral malleolar fractures Weber type A or B, either isolated or associated with medial or posterior malleolar fractures from January 2019 to August 2021. Patients with open fractures, associated injuries in the same limb, neuropathic or paralytic disorders, or associated syndesmotic injury were excluded.

Patients included in the study were randomized by even/odd numbers technique into two groups. **Group (A):** 40 patients had ORIF using lateral neutralization plate (ORIF group). **Group (B):** 40 patients had closed reduction and intramedullary screw fixation (IM screw group).

Patients who did not complete the 12-month follow-up period were excluded. At the end of this study, we had 73 included patients; 38 in group (A) and 35 in group (B).

The age of the patients ranged from 21 to 63 years with a mean value of 36.85 ± 12.13 years. There were 39 females (53.4%) and 34 males (46.6%) in the included patients. Twenty-seven patients had uni-malleolar fractures (37%), while 43 had bi-malleolar ankle fractures (58.9%). The other three patients sustained tri-malleolar ankle fractures (4.1%). Demographic data of included patients showed no significant difference between the two groups (Table 1).

**Table 1:** Demographic features of included patients

		**Group (A)** **ORIF**		**Group (B)** **IM screw**		***P*** **value**	**Total**	
		**Count**	**%**	**Count**	**%**		**Count**	**%**
**Sex**	**Male**	16	42.1%	18	51.4%	0.425	34	46.6%
	**Female**	22	57.9%	17	48.6%		39	53.4%
**Mode of trauma**	**Twisting injury**	26	68.4%	21	60.0%	0.706	47	64.4%
	**RTA**	6	15.8%	8	22.9%		14	19.2%
	**Direct fall**	6	15.8%	6	17.1%		12	16.4%
**Affected side**	**Right**	12	31.6%	12	34.3%	0.806	24	32.9%
	**Left**	26	68.4%	23	65.7%		49	67.1%
**History of diabetes**	**Present**	8	21.1%	7	20.0%	0.911	15	20.5%
	**Not**	30	78.9%	28	80.0%		58	79.5%
**Smoking**	**Smokers**	10	26.3%	10	28.6%	0.829	20	27.4%
	**Non-smokers**	28	73.7%	25	71.4%		53	72.6%
**Weber classification**	**Type A**	7	18.4%	7	20.0%	0.864	14	19.2%
	**Type B**	31	81.6%	28	80.0%		59	80.8%
**Fracture pattern**	**Transverse**	7	18.4%	7	20.0%	1	14	19.2%
	**Oblique**	27	71.1%	25	71.4%		52	71.2%
	**Comminuted**	4	10.5%	3	8.6%		7	9.6%

All patients included in the study received all the information about the procedure and randomization system and signed informed consent before surgery.

### Surgical technique

Surgery was performed after one to five days (average = 2.1 days) waiting for oedema subsidence. All patients were operated on under spinal anaesthesia. One gram of third-generation cephalosporins was given with induction of anaesthesia, and the course was continued for 24 hours post-operatively. Patients were positioned supine on a standard radiolucent operation table, under image intensifier guidance. A pneumatic tourniquet was placed on the thigh and inflated in group **A** patients and patients with associated medial malleolar fractures.

#### Group (A): ORIF group

**Fig. 1:**
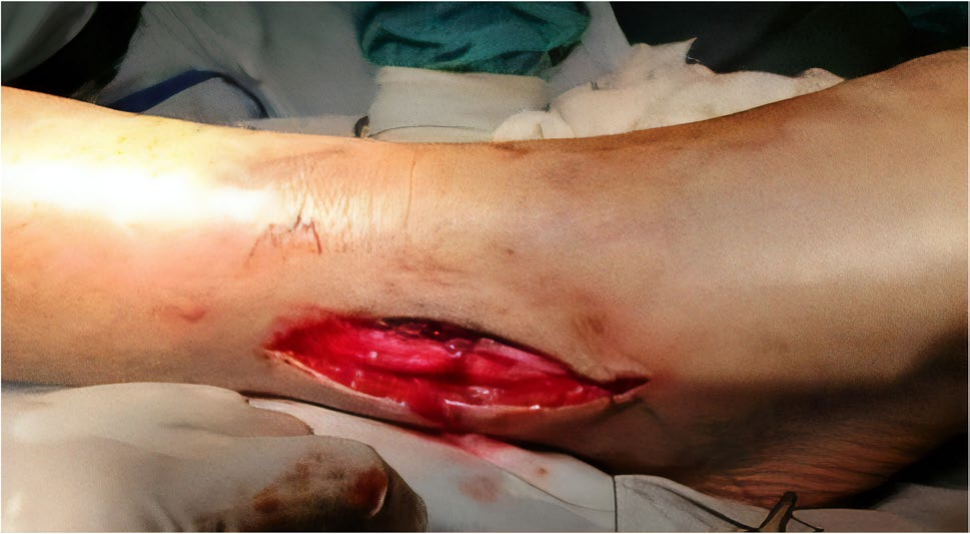
The skin incision over lateral malleolus for plate fixation

A straight incision was made over the lateral malleolus (Fig. 1). Then, blunt dissection through subcutaneous fat was made to avoid injury to the superficial peroneal nerve. The periosteum was dissected away to allow clean inspection of the fracture fragments.

**Fig. 2:**
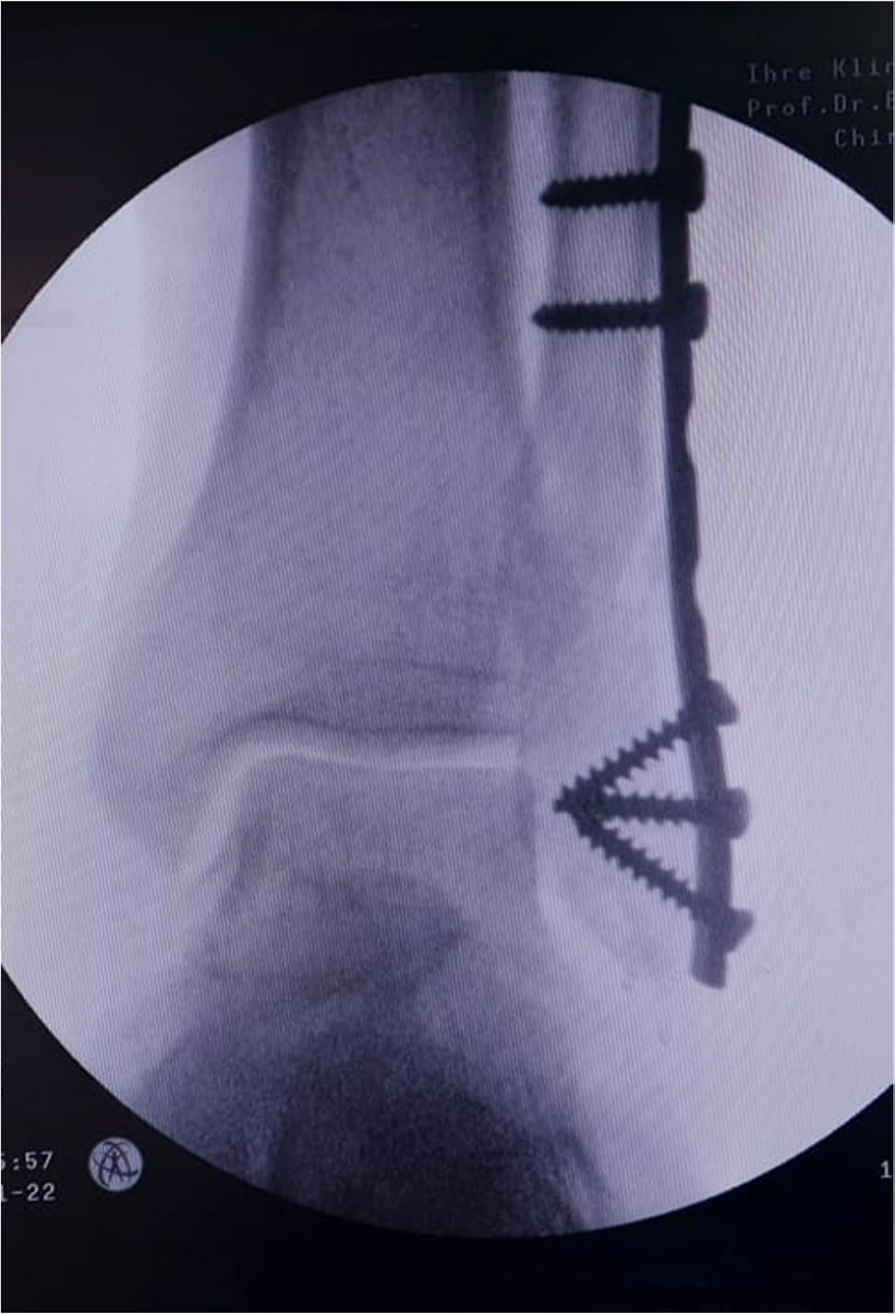
Intra-operative radiographic view after plate fixation

After the fracture was reduced, a pre-contoured one-third tubular plate was applied to the lateral side of the lateral malleolus and held to the bone by a plate holder. The plate was secured to the bone by three cortical screws above the fracture site and three cancellous screws below it (Fig. 2). Wounds were then closed in layers before adding a sterile gauze dressing.

#### Group (B): Intramedullary screw group

**Fig. 3:**
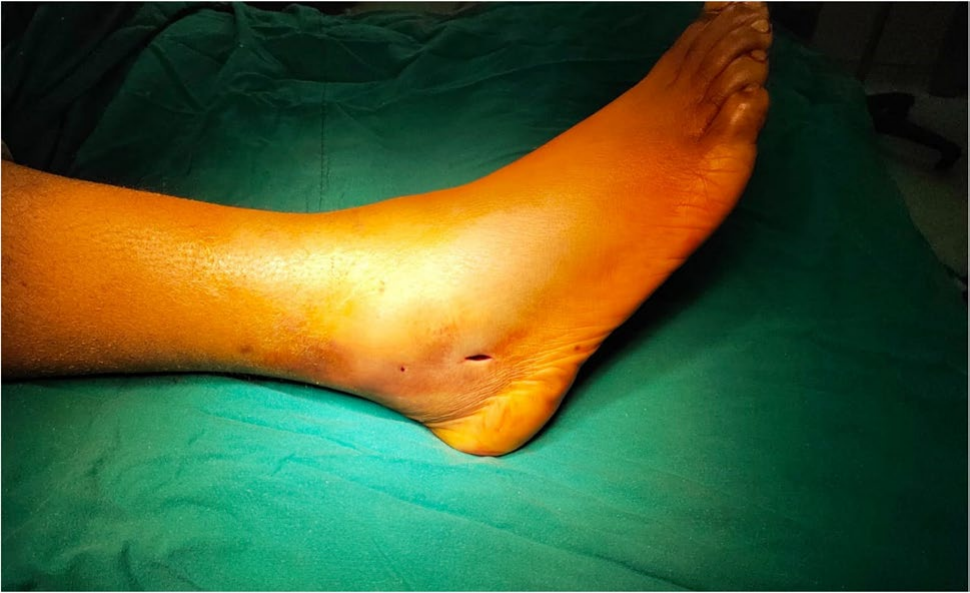
The incision made distal to the tip of lateral malleolus for screw insertion

**Fig. 4:**
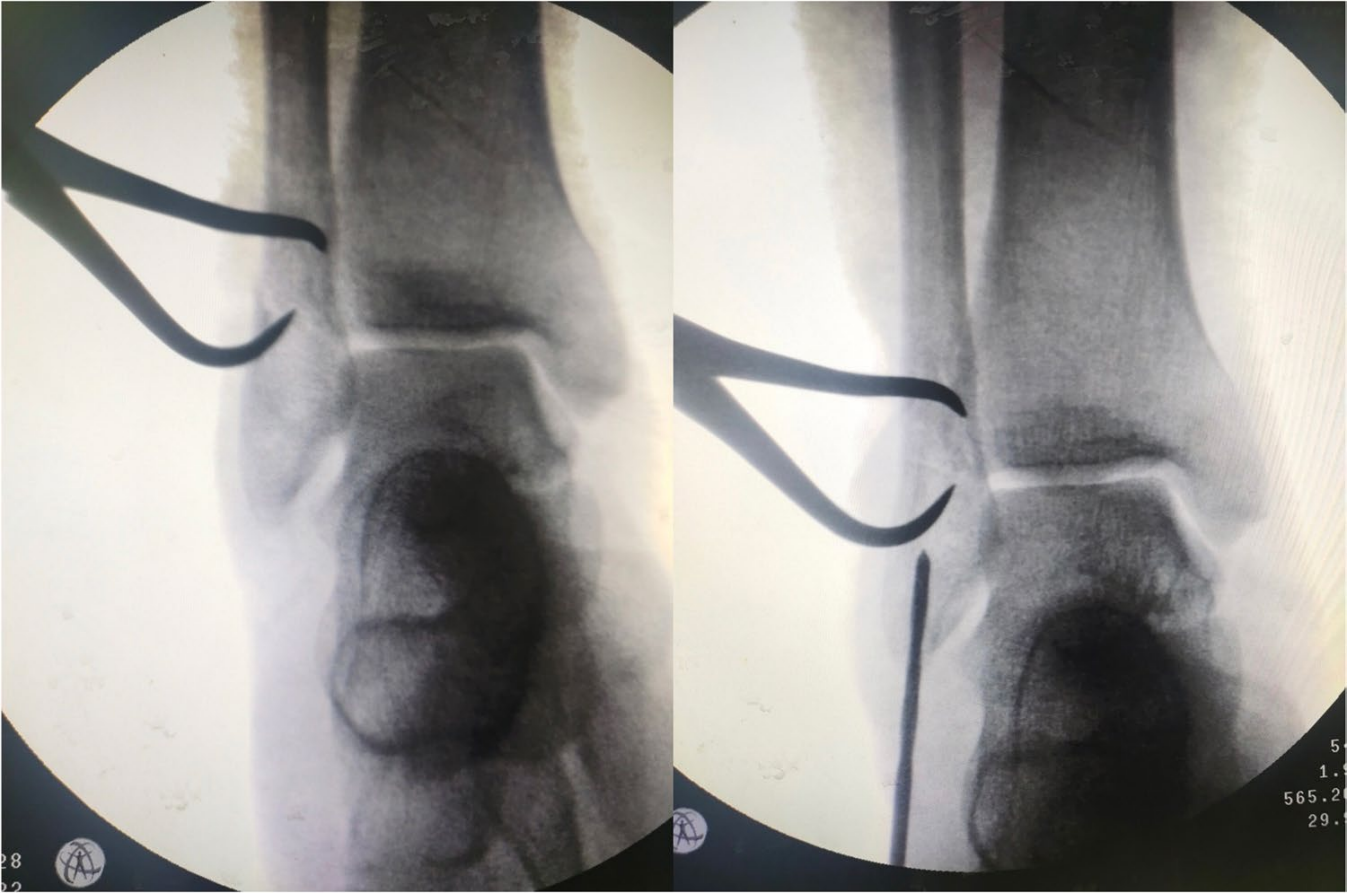
Achieving reduction with a pointed reduction clamp and making an entry point using a 2.5-mm drill bit

**Fig. 5:**
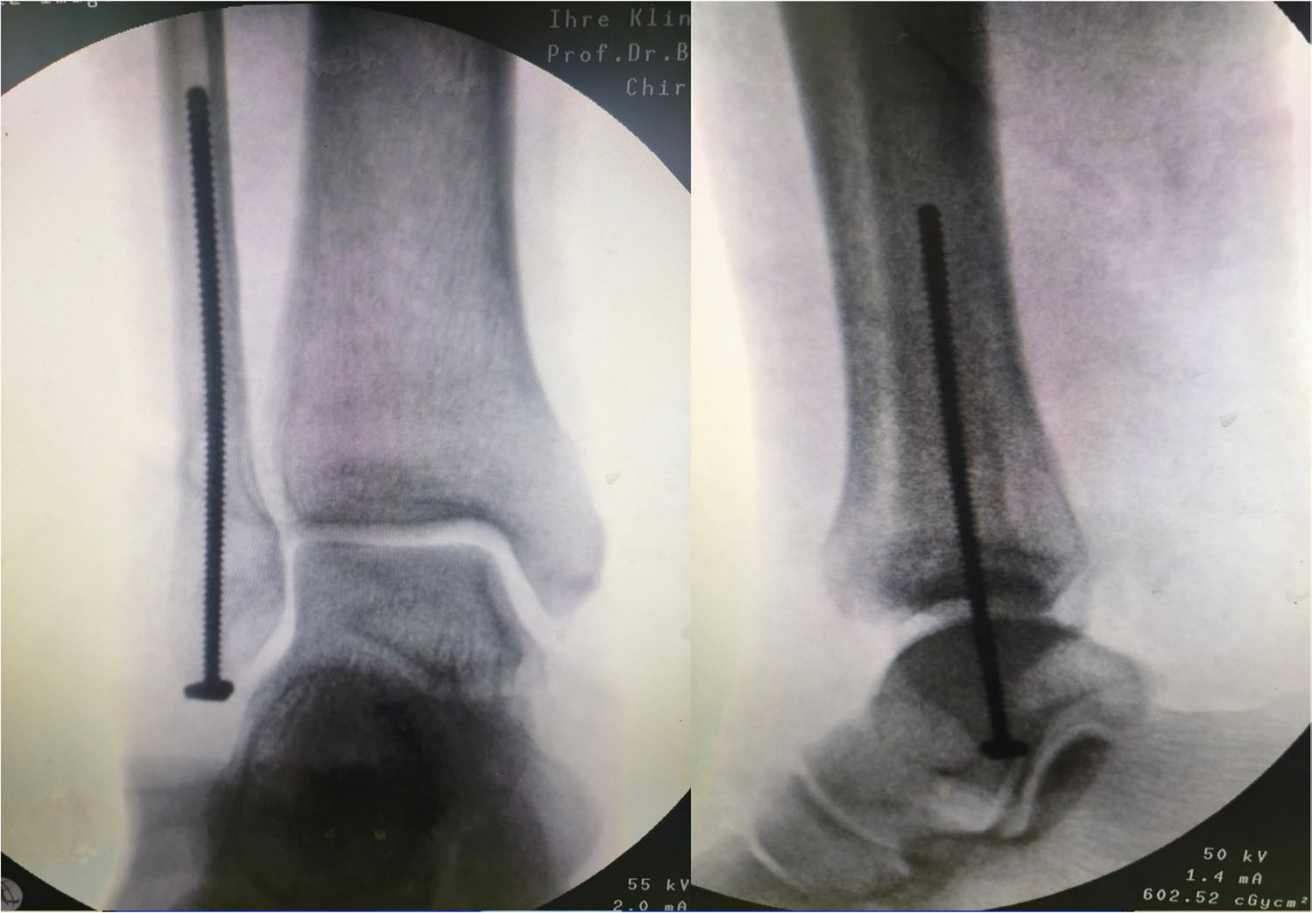
Insertion of the intramedullary screw

Under image intensifier guidance, closed reduction was achieved by inverting and internally rotating the foot while traction was applied. Then, reduction was maintained with a percutaneous pointed reduction forceps. A 1–2-cm incision was made from the tip of the lateral malleolus aiming distally and slightly posterior (Fig. 3). A 2.5-mm drill bit was used through the distal part (Fig. 4). Then, a 3.5-mm cortical, fully threaded screw with a washer was advanced until the washer reaches the bone (Fig. 5). The screw length varied from 90 to 110 mm.

Fixation of associated medial malleolar fractures was done using screws in 26 cases (60.5%), tension band wiring in five cases (11.6%), and anti-glide plate in 12 cases (27.9%). Posterior malleolar fractures in two out of three cases were fixed using percutaneous posterior to anterior screws.

### Post-operative management for both groups

The reduction was evaluated by post-operative radiographs using Mclennan J.G. and Ungersma scale [10]. Patients attended the clinic after two weeks for sutures removal. Then, all patients were followed both clinically and radiologically at regular visits every two weeks until full union was achieved. A below-knee slab was applied for four weeks before starting ankle range of motion. Partial weight-bearing was allowed after six weeks, and full weight-bearing was initiated when complete union was confirmed clinically and radiologically.

All patients were followed up for 12 months at least, with an average follow-up time of 12.7 months. The longest follow-up period was 17 months. The functional outcome was evaluated at the last visit using the American Orthopedic Foot and Ankle Society Score (AOFAS Ankle-Hindfoot Score) and modified Olerud and Molander Score (OMS).

Data were summarized using the mean and standard deviation or count and percentages. Comparisons were done using unpaired *t* test or chi-square *tests. *P* values < 0.05 were considered as statistically significant. SPSS 28 was used.

## Results

### Operative data

The mean operative time for all cases was 33.7 ± 5.56 minutes. It was significantly less in group (B); the IM screw group (28.66 ± 3.04) min when compared to group (A); the ORIF group (38.34 ± 2.34) min (*P*<0.001). The average size of the incision was significantly less in the intramedullary fixation group (1.9 cm) when compared to the lateral plate group (8.7 cm) (*P*<0.001).

Although there was relatively better adequacy of reduction in the ORIF group, it was statistically insignificant (*P*=0.504) (Table 2).

**Table 2:** Adequacy of reduction and functional outcome in both groups

		**Group (A)** **ORIF**		**Group (B)** **IM screw**		***P*** **value**	**Total**	
		**No.**	**%**	**No.**	**%**		**No.**	**%**
Adequacy of reduction *	Good*	36	94.7%	31	88.6%	0.504	67	91.8%
	Fair*	2	5.3%	3	8.6%		5	6.8%
	Poor*	0	0.0%	1	2.9%		1	1.4%
**Modified OMS grading**	**Excellent (90–100)**	26	68.4%	22	62.9%	0.705	48	65.8%
	**Good (70–89)**	10	26.3%	9	25.7%		19	26.0%
	**Fair (50–69)**	2	5.3%	4	11.4%		6	8.2%

*Adequacy of reduction

Good: No fibular shortening, < 2-mm posterior displacement, < 1-mm increase in medial clear space

Fair: 1–2-mm fibular shortening, 2–4-mm posterior displacement, 1–3-mm increase in medial clear space

Poor: > 2-mm fibular shortening, > 4-mm posterior displacement, > 3-mm increase in medial clear space

### Functional assessment

According to the (AOFAS) score, the mean score for group (A) was 87.11 ± 6.74. It was not significantly different from that in group (B) (86.57 ± 7.17) (*P*=0.573).

Functional outcome grading according to the modified OMS system showed no significant difference between both groups (*P*=0.705) (Table 2). The mean score in group (A) was 87.76 compared to 86.43 in group (B).

### Radiological assessment

A significant difference was present between both groups regarding the average time to full union as it was 9.11 weeks (range 7–16) in group (A) and 8.11 weeks (range 7 to 12) in group (B) (*P*=0.006).

### Complications

Both groups had no statistically significant difference in the incidence of certain complications such as superficial wound infection, deep infection, delayed union (≥ 16 weeks), or malunion. But there was a statistically significant difference in the incidence of hardware prominence as it was experienced in 12 cases in group (A) (31.6%) and was not present in group (B) (Table 3).

**Table 3:** The incidence of complications in each studied group

	**Group (A)** **ORIF**		**Group (B)** **IM screw**		***P*** **value**	**Total**	
	**No.**	**%**	**No.**	**%**		**No.**	**%**
**Superficial infection**	4	10.5%	1	2.9%	0.359	5	6.8%
**Deep infection**	1	2.6%	0	0.0%	1	1	1.4%
**Delayed union**	1	2.6%	0	0.0%	1	1	1.4%
**Malunion**	0	0.0%	1	2.9%	0.479	1	1.4%
**Hardware prominence**	12	31.6%	0	0.0%	< 0.001	12	16.4%

A second surgical procedure was needed in six patients in group (A): for debridement of deep infection in one case and removal of symptomatic hardware after complete union in the other five cases. Meanwhile, there was no need for second operations in group (B) patients.

## Discussion

The main concern during surgical treatment of lateral malleolar fractures is to achieve stable anatomic reduction and fixation. This is because any displacement in the lateral malleolus is followed by a talar shift [14, 15].

Several methods of fixation are present. The most common is the buttress plate, especially if comminuted or associated with syndesmotic injury [16]. Intramedullary fixation of lateral malleolus has several advantages including minimal soft-tissue dissection, short operative time, better healing with a short time of rehabilitation because of the minimally invasive nature of the procedure [12, 13].

Theoretically, intramedullary screws lack the rotational stability that can be provided by plate and screws construct and by fibular nails with locking screws [10, 17]. However, Bankston et al., in their biomechanical study on cadaveric modules with Weber type B-like fractures, proved equal resistance to torsional stresses in both intramedullary screw fixation and plate/lag screw construct [18]. In our series, we did not experience rotational instability during radiographic follow-up of cases with intramedullary screw fixation until full union, which can be explained by the post-operative immobilization in a back slab in the first four weeks, and the three-point contact achieved by the long screw within the fibular medullary canal.

Due to the narrow diameter of the distal fibula, intramedullary fixation was described in several studies using a single intramedullary device with variable diameters ranging from 2.0-mm smooth Steinmann pins up to 6.5-mm Acutrak plus compression screw (APCS) [12, 19–21]. The flexibility of the long 3.5-mm screw used (90–110 mm) allowed it to accommodate the bow of the distal fibular segment which was helpful to achieve the three-point contact within the medulla [22, 23].

In this study, 73 patients with fractures of the lateral malleolus were managed by either ORIF with a lateral plate or closed reduction and IM fixation with 3.5 fully threaded and self-tapping screw. The average follow-up period was 12.7 months. Groups were comparable as there was no significant difference in demographic characteristics. The adequacy of reduction revealed a good reduction in 94.7% of patients fixed with plates compared to 88.6% of patients fixed with intramedullary screws with no statistical significance (*P*=0.504). However, the average operative time and the wound size were significantly less in the intramedullary screw group compared to the plate fixation group.

The average time to union generally was 8.6 weeks. It was significantly longer in the ORIF group (9.11 weeks) versus (8.11weeks) in the intramedullary screw group. According to the modified OMS score, 94.7% of patients in the ORIF group had excellent to good outcomes compared to 88.6% in the IM screw group. No statistically significant difference was found in AOFAS. The complication rate was less in the intramedullary screw group compared to the ORIF group. Wound complications were encountered in 15.8% of patients in the ORIF group compared to 2.9% in the IM screw group. Symptomatic hardware was experienced exclusively in the ORIF group in 31.6% of cases.

We compared the results of our series with other studies. White et al. conducted a study on 125 patients with a mean age of 42 years having unstable ankle fractures treated with either ORIF using a plate (62 cases) or fibular nail (63 cases) [24]. Lee et al. retrospectively reviewed 23 patients with a mean age of 37.4 years having AO type-B2 ankle fractures fixed with Acutrak Plus Compression Screw (APCS) [21]. Latif et al. conducted a retrospective study on 46 patients with a mean age of 39.5 years with Weber A and B lateral malleolar fractures, fixed with intramedullary 3.5-mm screw [25]. Asloum et al. conducted their study on 60 patients with lateral malleolar fractures with a mean age of 53 years fixed with either plate (32 cases) or intramedullary nail (28 cases) [26]. The average follow-up period in our series was 12.7 months compared to 12.4 months in Lee’s study and 14.5 months in Latif’s series [21, 25].

The average wound size in Lee’s series with APCS fixation was 4.1 cm. In our study, we had a smaller average wound size when a 3.5-mm fully threaded intramedullary screw was used (1.9 cm). In the ORIF group, it was 8.7 cm [21].

The mean operative time in the IM screw group in our study was 28.7 minutes which was comparable to that in Lee’s study (25.3 min). In contrast, it was longer in the plate group in our study (38.3 min) [21].

Better reduction is usually associated with plate fixation. In the plate group in our series and White’s series, a good reduction was found in 36 cases (94.7%) and 60 cases (96.8%) respectively. When compared to the intramedullary fixation in both studies, good reduction could be obtained in 31 cases (89%) and 58 cases (92.1%) respectively. According to Latif et al., a good reduction was achieved in 43 cases (93.5%) fixed with intramedullary screws. Lee found a good reduction in 22 out of 23 cases (95.7%) and a fair reduction in one case (4.3%) [21, 24, 25].

The average time to union was 9.11 weeks in the plate fixation group compared to 8.11 in the IM screw group in our series. The latter was almost the same as in IM screw fixation in Latif and Ray’s studies (8.2 weeks) [23, 25].

According to OMS functional score; its mean value in Asloum’s series was 97 versus 83 (intramedullary nail versus plate), with a significant difference between both groups. In the ORIF group in our series, the score was 87.76 which was not significantly different from that in the IM screw group (86.43). Also, White found no significant difference between the scores in the IM nail and plate groups (78.4 versus 80.2 respectively) [24, 26].

Excellent to good outcome was obtained in 94.7% of cases in the plate group in our series compared to 88.6% in the IM screw group. According to Lee, excellent to good scores were found in 21 out of 23 cases (91.3%) compared to 97.8% of cases reviewed in Latif’s series. Ray had a lower rate of excellent to good outcome after a one year follow-up (84.2%) [21, 23, 25].

The most common complication usually encountered in plate fixation cases is symptomatic hardware. Brown reported that 39 out of the 126 (31%) patients with lateral malleolar fractures internally fixed with plates had lateral ankle pain related to the site of the plate. Symptomatic hardware was experienced in 66% of cases reviewed by Jacobsen et al. following plate fixation. Plate removal was done for 75% of them. The same findings were present in Tornetta and Creevy’s series in 56% of the cases. In this study, 12 out of 38 (31.6%) patients with lateral plating experienced symptomatic prominent hardware. Removal was needed in five cases after the failure of conservative measures. In contrast, White found a higher need to remove hardware with the fibular nail cases compared to plate fixation (19% versus 9.7%) [3, 24, 27, 28].

Infection may be another problem with a higher incidence with plate fixation. It was experienced in five cases (15.8%) in the ORIF group compared to one case (2.9%) in the IM screw group. Similarly, it was seen in nine cases (14.5%) compared to 2 cases (3.2%) in White’s study (plate vs fibular nail) [24].

The main concern of this study was to evaluate the clinical and radiographic outcomes of intramedullary screw versus plate fixation. Biomechanical analysis of fibular fractures has been addressed in several studies. Bankston et al. conducted a biomechanical study for comparing the use of intramedullary screw with buttress plate and lag screw when subjected to torsional loads. The intramedullary screw had 66.5% of the bone strength compared to 61.5% exhibited with plates, proving that the intramedullary screw is a stable fixation method [18].

Finite element analysis (FEA) modeling has not yet been used widely in the evaluation of different fixation methods in lateral malleolar fractures. Afandi et al. used FEA to compare using one-third tubular plates with locking compression plates in lateral malleolar fractures. Three or five screws were used, and they concluded that the optimum stability of one-third tubular plate was found while using five screws and that of locking compression plate was found while using three screws [29]. In another biomechanical study conducted by Marvan et al., FEA was used to evaluate the stability of several fixation techniques on distal fibular fractures. They found that among different modules compared in their study, the highest stability can be obtained by using an unlocking plate with six screws together with a lag screw while the lowest was to use an unlocking plate with four screws together with a lag screw [30].

Being a prospective randomized trial on a large sample size and inclusion of clinical and radiographic parameters in evaluation are of the strengths of this study. One of the limitations in this series was the inclusion of bi-malleolar and tri-malleolar fractures which may have influenced the results. Further studies may need to be focused on uni-malleolar fractures. Also, the biomechanical characteristics of both fixation methods need to be furtherly evaluated through future biomechanical studies and FEA modeling.

## Conclusion

The use of intramedullary screw fixation is an efficient and safe alternative to the classic ORIF methods using neutralization plates in unstable low lateral malleolar fractures. Plate fixation may be more suitable to achieve anatomical reduction. However, percutaneous intramedullary screw fixation is associated with fewer complication rates, especially in wound problems and symptomatic hardware. So, it may be used more often whenever acceptable closed reduction of the fracture can be achieved, in elderly patients and those with chronic comorbidities who are more likely to develop wound complications.
